# Fitness of *Mycobacterium tuberculosis* Strains of the W-Beijing and Non-W-Beijing Genotype

**DOI:** 10.1371/journal.pone.0010191

**Published:** 2010-04-16

**Authors:** Andrea von Groll, Anandi Martin, Matthias Stehr, Mahavir Singh, Françoise Portaels, Pedro Eduardo Almeida da Silva, Juan Carlos Palomino

**Affiliations:** 1 Mycobacteriology Unit, Institute of Tropical Medicine, Antwerp, Belgium; 2 Department of Genome Analysis, Helmholtz Centre for Infection Research, Braunswcheig, Germany; 3 Laboratory of Mycobacteriology, Universidade Federal do Rio Grande, Rio Grande, Brazil; University of California Merced, United States of America

## Abstract

**Background:**

Multidrug resistant tuberculosis (MDR-TB) is a major threat for global tuberculosis control. The W-Beijing *Mycobacterium tuberculosis* genotype has been associated with drug resistance. Elucidation of the mechanisms underlying this epidemiological finding may have an important role in the control of MDR-TB. The aim of this study was to evaluate the fitness of drug-susceptible and MDR *M. tuberculosis* strains of the W-Beijing genotype compared with that of Non-W-Beijing strains.

**Methodology/Principal Findings:**

Fitness of *M. tuberculosis* strains was determined by evaluating the difference in the growth curves obtained in the MGIT960 automated system and assessing the competitive growth capacity between W-Beijing and non-W-Beijing strains. The W-Beijing MDR strains had a significant longer lag phase duration compared to the other strains but did not present a significant fitness cost. When grown in competition they had an advantage only in medium containing 0.1% Tween 80.

**Conclusions/Significance:**

It was not possible to confirm a selective advantage of W-Beijing strains to grow, except for differences in their resistance to Tween 80. Further studies are needed to elucidate the putative advantage of W-Beijing strains compared to other genotypes.

## Introduction

According to the World Health Organization (WHO), there were globally an estimated 9.27 million new cases of tuberculosis (TB) and 1.3 million deaths in 2007 [1]. Multidrug resistant tuberculosis (MDR-TB), caused by *Mycobacterium tuberculosis* strains resistant to at least rifampicin (RMP) and isoniazid (INH), is a major threat for global TB control with direct effect on the epidemiology of the disease.

Molecular epidemiological tools and genotyping of *M. tuberculosis* strains have identified different families and genotypes contributing to a better knowledge of the transmission dynamics of TB and identification of certain genotypes more widespread than others [2]. The W-Beijing genotype has been strongly associated with drug resistance in several settings [3]–[6] and in regions with high incidence of MDR-TB [7]. The mechanisms underlying this epidemiological finding have not been clearly elucidated and its knowledge may have an important role in the control of MDR-TB.

Some explanations have been advanced but they have not been conclusive. The W-Beijing genotype could have an enhanced ability to acquire drug resistance due to a polymorphism in genes coding for DNA repair enzymes [8]. However, W-Beijing strains have shown similar rates of mutation-conferring resistance to RMP compared to non-W-Beijing strains suggesting that they do not mutate more frequently than non-W-Beijing strains [9]. W-Beijing strains could offset the physiological cost that would be expected from the acquisition of drug resistance and thus, spread in the population with the same efficiency as the susceptible ones. However, comparison of fitness between in vitro *rpoB* mutants showed that W-Beijing and non-W-Beijing strains had similar biological cost for the same *rpoB* mutation [10].

Although there are conflicting results between epidemiological and laboratorial studies, some important experimental findings have contributed to understand and explain epidemiological situations. While molecular epidemiological studies have shown that there is predominance and successful transmission of specific mutations, such as Ser315Thr in *katG* and Ser531Leu in *rpoB*, associated with MDR *M. tuberculosis* in several populations [11]–[14], laboratorial experiments found that those mutations cause a lower biological cost in the bacteria [10], [15], [16]. Furthermore, the fitness was also dependent on the genetic background of the strain, which can develop compensatory mutations to mitigate the biological cost [17].

There are few fitness studies of *M. tuberculosis* taking into account the genetic background of the strains. This is mainly due to the limited availability of methods to compare fitness of the bacteria. In this study we have comparatively evaluated the fitness of drug-susceptible and MDR *M. tuberculosis* strains belonging to the W-Beijing genotype with that of non-W-Beijing strains. Fitness of *M. tuberculosis* was determined by different methods that assess the competitive growth capacity of the strains and evaluate differences in the growth curves obtained with the MGIT960 automated system.

## Results

### Study of fitness by growth curves

**Figure 1:**
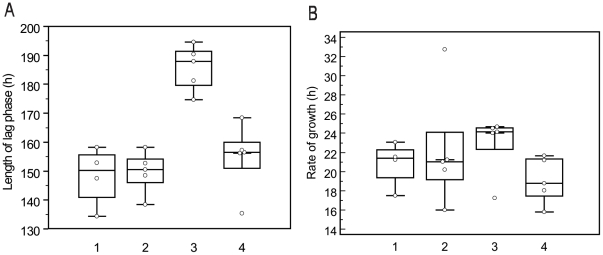
Comparison of the fitness between W-Beijing and non-W-Beijing strains. The length of lag phase (Fig. 1A) and rate of growth (Fig. 1B) were assessed for strains W-Beijing drug susceptible (Group 1), non-W-Beijing drug susceptible (Group 2), W-Beijing multidrug resistant (Group 3) and non-W-Beijing multidrug resistant (Group 4). The average length of lag phase for groups 1, 2, 3 and 4 were 148.3 h, 149.7 h, 185.8 h and 154.7 h, respectively. Group 3 presented the longest length of lag phase compared to the other three groups (*P*<0.001). The average rate of growth for groups 1, 2, 3 and 4 was 20.8 h, 22.2 h, 22.9 h and 19.1 h, respectively, but the difference among the groups was not statistically significant (*P* = 0.462).

As shown in Table 1 four strains were W-Beijing drug susceptible (Group 1), five strains non-W-Beijing drug susceptible (Group 2), five strains W-Beijing MDR (Group 3) and five strains non-W-Beijing MDR (Group 4). We found that the average length of lag phase for groups 1, 2, 3 and 4 were 148.3 h, 149.7 h, 185.8 h and 154.7 h, respectively (Figure 1A). Group 3 corresponding to W-Beijing MDR strains presented the longest length of lag phase (*P*<0.001). The average rate of growth for groups 1, 2, 3 and 4 was 20.8 h, 22.2 h, 22.9 h and 19.1 h, respectively (Figure 1B) but the difference was not statistically significant (*P* = 0.462).

**Table 1:** Study of fitness by growth curve: length of lag phase and rate of growth of each strain.

	Strain Number	Geographic Origin	Genotype by PCR	Profile of susceptibility	Length of lag phase	Rate of growth
Group 1	02-2761	Bangladesh	W-Beijing	S	152,86	21,23
	08-0774	Sweden	W-Beijing	S	147,50	23,05
	09-1051	Argentina	W-Beijing	S	158,30	17,50
	09-1029	Argentina	W-Beijing	S	134,40	21,54
Group 2	09-0428	Demo Rep Congo	non-W-Beijing	S	148,50	21,22
	01-2522	Georgia	non-W-Beijing	S	150,46	21,02
	09-0168	Demo Rep Congo	non-W-Beijing	S	158,28	20,19
	09-0365	Tanzania	non-W-Beijing	S	152,70	15,99
	09-0009	Demo Rep Congo	non-W-Beijing	S	138,46	32,77
Group 3	08-1990	Demo Rep Congo	W-Beijing	MDR	194,64	24,68
	03-0265	Bangladesh	W-Beijing	MDR	174,74	24,13
	07-2983	Thailand	W-Beijing	MDR	190,37	24,54
	07-3088	Georgia	W-Beijing	MDR	187,95	17,25
	07-3214	Georgia	W-Beijing	MDR	181,28	24,02
Group 4	08-1585	Demo Rep Congo	non-W-Beijing	MDR	156,16	15,78
	08-1421	Nigeria	non-W-Beijing	MDR	157,25	18,02
	08-1790	Bangladesh	non-W-Beijing	MDR	135,34	21,18
	03-0850	Georgia	non-W-Beijing	MDR	168,40	18,79
	08-1302	Bangladesh	non-W-Beijing	MDR	156,55	21,67

S: susceptible to first and second-line drugs tested; MDR: multidrug resistant strain. Demo Rep Congo: Democratic Republic of Congo.

### Characterization of *M. tuberculosis* strains selected for competitive growth

Strains 02-2761 and 03-0265 were confirmed as W-Beijing and according to SITVIT database [18] had the ST 01 profile; strains 01-2522 and 03-0850 were classified as T1, and strains 01-2522 and 03-0850 had the ST 156 and ST 264 profiles, respectively. The two MDR strains had the mutation Ser315Thr in *katG* associated with resistance to INH and Ser531Leu in *rpoB* associated with resistance to RMP.

### Fitness studies by competitive growth methodology

Table 2 shows results of the four competition studies with number and percentage of viable cells before and after competition and the number of generations (n) for each strain. The strain 03-0265 (W-Beijing, MDR) had the lowest growth of all strains (n = 3.8) in competition with the strain 02-2761 (W-Beijing, S), but its growth increased (n = 7.4) in competition with the strain 03-0850 (non-W-Beijing MDR). The strain 01-2522 (non-W-Beijing, S), had a stable and low growth in competition (n = 5.2) with the strain 03-850 (non-W-Beijing MDR) and (n = 5.5) with strain 02-2761 (W-Beijing, S). This strain 02-2761 showed a growth of n = 8.2 and n = 11.6 after competition with the strains 03-0265 and 01-2522, respectively. The strain 03-850 (non-W-Beijing MDR) had a higher growth in competition with strains 03-0265 (n = 14.2) and 01-2522 (n = 11.2).

**Table 2:** Viable cells (concentration and percentage) of *M. tuberculosis* before and after competition assay between resistant and susceptible strains with the same genotype family.

	Profile of susceptibility	Genotype	Strain	Before competition		After competition		Number of Generation (n)
				CFU/mL	%	CFU/mL	%	
Test 1	MDR	W-Beijing	03-0265	3.86×10^4^	9.5%	5.2×10^5^	0.5%	3.8
	S	W-Beijing	02-2761	3.68×10^5^	90.5%	10.7×10^7^	99.5%	8.2
Test 2	MDR	Non-W-Beijing	03-0850	2.23×10^4^	7.0%	5.2×10^7^	82%	11.2
	S	Non-W-Beijing	01-2522	2.97×10^5^	93%	1.11×10^7^	18%	5.2
Test 3	MDR	W-Beijing	03-0265	3.86×10^4^	63.38%	6.60×10^6^	1.57%	7.4
	MDR	Non-W-Beijing	03-0850	2.23×10^4^	36.32%	4.13×10^8^	98.43%	14.2
Test 4	S	W-Beijing	02-2761	3.68×10^5^	55.34%	1.11×10^9^	98.82%	11.6
	S	Non-W-Beijing	01-2522	2.97×10^5^	44.66%	1.32×10^7^	1.17%	5.5

S: susceptible to first and second-line drugs tested; MDR: multidrug resistant strain.

### Fitness studies assessed by qPCR

Efficiency of the reaction determined with the nBj and Bj primers was 1.86 and 1.96, respectively, showing that both had good efficiency for determination of relative quantification. Table 3 shows the ratio of genomic DNA of the non-W-Beijing strain compared to W-Beijing. For each test the results show the ratio found in the starting inoculum (DNA extracted at time 0 of the competition), the ratio after competition in the presence and absence of Tween 80 and the ratio of the same strains growing alone. Test 5 showed that strain 03-0850 (MDR-Non-W-Beijing) had an inoculum ratio of 6.4 compared to 03-0265 (MDR-W-Beijing); after competition increased to 14.2 but in the presence of Tween 80 the ratio was only 2.5. Test 6 showed that strain 01-2522 (S-Non-W- Beijing) had an inoculum ratio of 2.1 in relation to strain 02-2761 (S-W-Beijing). After competition the ratio decreased to 1.7 in 7H9 and in the presence of the Tween 80 the ratio was 0.01. Test 7 showed strain 01-2522 had an inoculum ratio of 2.8 in relation to strain 03-0265 after competition decreased to 2.4 in 7H9 and in the presence of Tween 80 the ratio was 0.01. Test 8 found strain 03-0850 had an inoculum ratio of 5.6 in relation to strain 02-2761 and after competition decreased to 5.1 in 7H9; with Tween 80 the ratio was 0.8.

**Table 3:** Ratio of the DNA genomic determined by qPCR between the strains non-W-Beijing in relation to W-Beijing before and after competition.

	Profile of susceptibility	Genotype	Strain	Inoculum Ratio	7H9		7H9 + Tween	
					Competition ratio	Alone growth ratio	Competition ratio	Alone growth ratio
Test 5	MDR	Non-W-Beijing	03-0850	6.4	14.2	3.2	2.5	0.4
	MDR	W-Beijing	03-0265					
Test 6	S	Non-W-Beijing	01-2522	2.1	1.7	6.7	0.01	0.002
	S	W-Beijing	02-2761					
Test 7	S	Non-W-Beijing	01-2522	2.8	2.4	7.5	0.01	0.004
	MDR	W-Beijing	03-0265					
Test 8	MDR	Non-W-Beijing	03-0850	5.6	5.1	2.7	0.8	0.85
	S	W-Beijing	02-2761					

S: susceptible to first and second-line drugs tested; MDR: multidrug resistant strain.

## Discussion

The use of growth curves based on metabolic activity has been applied before in fitness studies [19], [20]. The two parameters here obtained were the length of lag phase and the rate of growth. The only significant difference was found for W-Beijing MDR strains that had a longer lag phase duration. The lag phase is the period required to adapt to their new environment when actively synthesizing enzymes to metabolize novel nutrients. The strains used in this study had few sub-cultures after primary isolation; thus, it is possible that the resistant strains needed a longer period to adapt to the new medium without drugs. The rate of growth, was similar in the four groups studied. It is normally assumed that MDR strains present a biological cost for growth in the absence of drugs. The most common mechanism associated with the acquisition of resistance to RMP is a mutation in the *rpoB* gene [21] and for INH are mutations in *katG* and the *inhA* operon [22], [23]. These genes encode enzymes of biological significance in the bacteria, once they mutate, their function could be impaired leading to a fitness cost. Previous studies have shown that the biological cost could vary according to the mutation (aminoacid and position) and the strain genetic background. Also compensatory mutations could reverse the biological cost [10]. Thus, W-Beijing strains could be more efficient in restoring fitness due to mutations in DNA repair enzymes, which might provide a selective advantage [8]. This hypothesis, however, was not confirmed in the present study by comparison of the growth curves.

Another approach to assess fitness of the bacteria is by their competitive capacity to grow. Competitive tests are frequently performed to determine the relative fitness associated with a specific drug-resistance mutation [24]. This is carried out with one susceptible and one drug-resistant strain harbouring specific mutations. After growing in competition in a liquid medium they are plated in agar with or without antibiotics. The relative fitness is defined as the ratio of the generation rate between the resistant and susceptible strain [25]. We adapted this methodology to study the competitive capacity of W-Beijing and non-W-Beijing strains. Since both tested strains had the same drug susceptibility profile, differentiation was not possible by drug selection. We used PCR to differentiate W-Beijing and non Beijing strains from each colony obtained after competition. Since all strains belonging to the same group had similar metabolic activity in the MGIT960 system, we selected one strain from each group for the competitive study: the two strains previously classified as W-Beijing by PCR had the characteristic signature by spoligotyping and the two non-W-Beijing strains were typed as T1 genotype. Both MDR W-Beijing and non-W-Beijing strains had the same mutations: Ser531Leu in *rpoB* and Ser315Thr in *katG*. These mutations are the most frequently found in clinical settings and have also demonstrated a lower fitness cost in comparison to other mutations for RMP and INH in experimental studies [10], [14]–[16]. Again, we could not confirm a selective advantage of the MDR W-Beijing compared to MDR non-W-Beijing. On the contrary, the MDR non-W-Beijing strain had a growth advantage in competition. This could be explained by the growth profile found in MGIT960. Since the MDR W-Beijing presented a longer lag phase, the longer time to start growing could be a disadvantage in competition when there are fewer nutrients available. The intriguing result was the low efficiency of the non-W-Beijing susceptible strain (02-2522) to growth in competition. If we analyse its growth in MGIT960 it had the shortest lag phase and the second highest rate of growth, however, its growth was unsuccessful in competition. There were some different factors to consider about growth in competition related to MGIT960: the strains did not rely only on the capacity to growth in 7H9 liquid medium, but further on 7H10 agar, the presence of 0.1% Tween 80 added in 7H9 medium to obtain homogeneous cell suspensions and the growth in competition with another strain. To investigate this we performed a new competition test between W-Beijing and non-W-Beijing strains in the presence and absence of Tween 80. We applied qPCR to determine relative quantification of genomic DNA between the two strains before and after competition. In the presence of Tween 80 strain 02-2522 (susceptible, non-W-Beijing) grew poorly. Tween 80 is an ionic surfactant used frequently when growing *M. tuberculosis* in liquid medium. Effects of surfactants on the mycobacterial cell wall have been studied before. In *M. avium* it was found to decrease the quantity of glycolipids, which are virulent factors, in the cell wall increasing the permeability of antituberculosis drugs [26]. Different resistance to Tween among *M. tuberculosis* strains would be important to consider not only for its use in mycobacterial cultures, but related to the relevance of pulmonary surfactants having an important function as host defence mechanism. If *M. tuberculosis* strains have different susceptibility to pulmonary surfactants, and this could be associated with a specific genotype, it could represent differences in the cell wall with influence in the permeability of drugs or virulence factors.

Taken together these results show that it was not possible to confirm a selective advantage of W-Beijing strains to grow, except for differences in their resistance to Tween 80. These results reflect what occurs *in vitro*, being in this way limited to reproduce the *in vivo* interaction between pathogen and the host. Further studies of W-Beijing strains associated to pulmonary surfactants could give new insights for understanding factors inherent to the pathogen inside the host that could influence the course of the disease.

## Materials and Methods

### Study design

The study was performed in three phases: phase I involved comparison of fitness between W-Beijing and non-W-Beijing strains performing growth curves using the MGIT960 system; phase II studied fitness by assessing the competition between W-Beijing and non-W-Beijing strains by viable cell counting; and phase III assessed the competitive fitness using Real-Time PCR (qPCR).

### Strains

Nineteen *M. tuberculosis* strains of the W-Beijing and non-W-Beijing genotype with different drug susceptibility profiles were selected. Initial identification as W-Beijing or non-W-Beijing was performed by a polymerase chain reaction (PCR) assay previously described [27]. Drug susceptibility profile was determined by the proportion method according to standard procedures [28]. From the 19 strains, four were selected for the competitive fitness studies, one strain each of W-Beijing-MDR, W-Beijing drug susceptible, non-W-Beijing-MDR, and non-W-Beijing drug susceptible. Strains were fully characterized and analyzed for the presence of mutations conferring resistance to RMP and INH. Spoligotyping was performed with a commercial kit (Ocimum Biosolutions BV, India) according to standard procedures [29].

### Molecular characterization

Genomic DNA was obtained by resuspending a loopful of culture into 200 µl of TE buffer (10 mM Tris-HCl, 1 mM EDTA pH 8.0) heat-inactivated at 100°C for 10 min and centrifuged for 20 min at 10,000 x g at 4°C. DNA sequencing was performed to look for mutations in *rpoB*, *katG* and the *inhA* promoter associated with resistance to RMP and INH, respectively. Sequencing of *rpoB* was performed as previously described [30]. For INH the primers were TB86/TB87 to amplify *katG* and TB92/TB93 for the *inhA* promoter [31]. The PCR product was sequenced with an automatic DNA sequencer (Applied Biosystems 3730 DNA analyzer). The ClustalX program (version 1.83.1) and Genedoc software (version 2.100) were used to analyze the final nucleotide sequences.

### Phase I: Fitness studies by growth curve using the MGIT960 system

All strains were freshly subcultured on Löwenstein Jensen medium and incubated at 37°C for exactly 3 weeks. The inoculum was prepared by suspending bacilli in 4 ml ultra-pure water containing glass beads. The suspension was vortexed for 30 s and allowed to sediment for 15 min. The supernatant was transferred to another tube, diluted to match the turbidity of a McFarland tube No. 0.5 and adjusted at 595 nm to an OD of 0.01–0.03. A dilution 1∶10 was prepared in ultra-pure water. One hundred µl of this dilution was added in triplicate to MGIT Mycobacteria Growth Indicator Tubes (Becton Dickinson Diagnostic Systems, Sparks, MD, USA) supplemented with 10% MGIT960 SIRE™ Supplement (Becton Dickinson, USA). The tubes were entered into the MGIT960 system and incubated at 37°C. Growth curves were obtained by monitoring the fluorescence and recording the growth units (GU) every hour using the BD EpiCenter™ software.

### Growth parameters

The fitness of each strain was compared by two parameters from the growth curves: the length of the lag phase and the rate of growth. The length of lag phase was determined from the start of incubation until reaching 75 GU (positive threshold in MGIT960). The rate of growth was the time in hours necessary for a strain to increase from 5,000 to 10,000 GU in the MGIT960 system. It was calculated from the growth curve considering that all strains were in the logarithmic phase of growth between these two points.

Differences in these two parameters were calculated with ANOVA and Student-Newman-Keuls tests for all pairwise comparisons with the MedCal™ software (v9.6.2.0; Mariakerke, Belgium). It was considered significant if *P*<0.05.

### Phase II: competitive fitness determined by viable cell counting

In this experiment, the drug-susceptible and drug-resistant W-Beijing and Non-W-Beijing *M. tuberculosis* strains competed in a common environment.

Four selected *M. tuberculosis* strains: 03-0265 (W-Beijing-MDR), 02-2761 (W-Beijing-drug susceptible), 03-0850 (Non-W-Beijing-MDR) and 01-2522 (Non-W-Beijing - drug susceptible) were freshly subcultured on Löwenstein Jensen medium and incubated at 37°C. A bacterial suspension was transferred into separate tubes containing 5 mL of 7H9 medium (Middlebrook 7H9 medium with 0.1% casitone, 0.5% glycerol, 10% OADC (oleic acid, albumin, dextrose and catalase) (Becton-Dickinson, USA) and 0,1% Tween 80 and incubated at 37°C for 20 days. The turbidity of each tube was adjusted according to the less concentrated tube and further diluted 10^−1^ and 10^−2^.

Pairwise competition was performed as follows according to previously described methodology [25] tube 1, 03-0265 (W-Beijing-MDR) and 02-2761 (W-Beijing-drug susceptible); tube 2, 03-0850 (Non-W-Beijing-MDR) and 01-2522 (Non-W-Beijing - drug susceptible); tube 3, 03-0265 (W-Beijing-MDR) and 03-0850 (Non-W-Beijing-MDR); and tube 4, 02-2761 (W-Beijing-drug susceptible) and 01-2522 (Non-W-Beijing - drug susceptible). Each tube contained 4 mL of 7H9 medium, 0.1% Tween 80, 50 µL of the inoculum diluted 10^−1^ for the susceptible strains and 50 µl of the inoculum diluted 10^−2^ for the MDR strains. The number of viable cells in the inocula was estimated according to Miles and Misra [32]. Tubes were incubated at 37°C and after 17 days the number of colony forming units (CFU) in each tube was determined by plating serial 10-fold dilutions in Middlebrook 7H10 agar with or without RMP at 5 µg/ml for tube 1 (W-Beijing-MDR + W-Beijing-S) and tube 2 (Non-W-Beijing-MDR + Non-W-Beijing-S). CFUs in tube 3 (W-Beijing-MDR + Non-W-Beijing-MDR) and tube 4 (W-Beijing-S + Non-W-Beijing-S) was determined by plating serial 10-fold dilutions in Middlebrook 7H10 agar without RMP. All plates were incubated at 37°C. After 4 weeks the number of CFU of the drug-susceptible strain was calculated by subtracting the number of CFUs of the drug-resistant from the total number of CFU. For tubes 3 and 4 to differentiate and calculate the number of W-Beijing CFU from the non-W-Beijing CFU each colony was sub-cultured in Middlebrook 7H9 liquid medium and then, a PCR with specific primers for identification of the W-Beijing and non-W-Beijing genotypes was performed [27]. In order to compare the growth of each strain in competition, the number of generations was determinate using the formula:
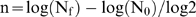



Whereas: n  =  number of generation; N_0_ =  initial number of viable cells; N_f_ =  final number of viable cells;

### Phase III. Competitive fitness quantified by qPCR

In this experiment, the fitness of W-Beijing and Non-W-Beijing strains with the same drug-susceptibility profile was assessed by qPCR.

The inoculum was prepared as described above for the phase II studies. The competition was performed in two sets of 8 tubes containing 5 ml of 7H9 medium with (set one) or without (set two) 0.1% Tween 80. Tube 1: 50 µl each of 03-0265 (W-Beijing-MDR) and 03-0850 (Non-W-Beijing-MDR); tube 2: 50 µL of 02-2761 (W-Beijing-drug susceptible) and 01-2522 (Non-W-Beijing - drug susceptible); tube 3: 50 µL of 03-0265 (W-Beijing-MDR) and 01-2522 (Non-W-Beijing - drug susceptible); tube 4: 50 µL of 02-2761 (W-Beijing-drug susceptible) and 03-0850 (Non-W-Beijing-MDR); tube 5: 50 µl of 03-0265 (W-Beijing-MDR); tube 6: 50 µl of 03-0850 (Non-W-Beijing-MDR); tube 7: 50 µL of 02-2761 (W-Beijing-drug susceptible); and tube 8: 50 µL 01-2522 (Non-W-Beijing - drug susceptible). DNA was extracted from all tubes at time 0 and incubated at 37°C for 30 days. One ml from each tube was used for DNA extraction after sedimentation at 5,000 x g for10 min and the pellet resuspended in Lysis Extraction Solution (50 mM Tris HCl pH 7.5, 10 mM EDTA, 0.5% SDS, 50 mM NaCl, 300 µg/mL Proteinase K). Purification was performed with chloroform-amyl alcohol (24∶1) and precipitation with 3 M Sodium Acetate and 500 µL of absolute ethanol. The DNA precipitate was washed with 75% ethanol and suspended in 50 µL of ultra pure water.

The relative quantification of W-Beijing and non-W-Beijing strains was performed by qPCR according to Hillemann et al. [33]. For each sample, two reactions were performed in triplicate, the first with primers nBjF: 5′-AAGCATTCCCTTGACAGTCGAA-3′ and nBjR: 5′-GGCGCATGACTCGAAAGAAG-3′ to quantify the non-W-Beijing strain and the second with primers BjF5′-CTCGGCAGCTTCCTCGAT-3′ and BjR5′-CGAACTCGAGGCTAGCCTACTAC-3′ to quantify W-Beijing. The reaction was performed in a final volume of 10 µL consisting of 1x QuantiTect SYBR Green PCR Kit (Qiagen), 0.33 µM of forward and reverse primers and 1 µL of a 1∶100 dilution of DNA. PCR was run in a LightCycler® 480 Real-Time PCR System (Roche) at 95°C for 15 min, 40 cycles of 95°C for 15 s, 55°C for 20 s, 72°C for 30 s and for the melting curve 95°C 5 s, 70°C 1 min 95.

DNA extracted from strains 03-0265 (W-Beijing) and 03-0850 (non-W-Beijing) were diluted 1∶2, 10^−1^, 10^−2^, 10^−3^, 10^−4^, 10^−5^ and 10^−6^ and tested in triplicate to determine the efficiency of the primers Bj and NBj. For determining the relative fitness, the LightCycler® 480 software calculated the proportion between the W-Beijing strain and non-W-Beijing taking into account the efficiency of the primers and the Threshold Cycle (TC) obtained for each strain in each reaction.
